# Investigating the Maternal Factors Associated with Preschool Children’s Food Preferences; A Cross-Sectional Study

**DOI:** 10.31661/gmj.v8i0.1652

**Published:** 2019-09-18

**Authors:** Nayyereh Farajzadeh-Moghanjoughi, Sorayya Kheirouri, Mohammad Alizadeh, Alireza Farsad-Naeimi

**Affiliations:** ^1^Department of Nutrition, Faculty of Nutrition and Food Sciences, Tabriz University of Medical Sciences, Tabriz, Iran; ^2^Department of Nutrition, Faculty of Medicine, Hamadan University of Medical Sciences, Hamadan, Iran

**Keywords:** Children, Food Preferences, Mothers, Knowledge, Attitude

## Abstract

**Background:**

Dietary patterns of children are determined by their food preferences, and mothers have important implications for these preferences. This study was aimed to investigate the maternal factors associated with children’s food preferences.

**Materials and Methods:**

In this cross-sectional study, a total of 576 healthy children aged 3-6 years participated from nursery schools through simple cluster sampling method and maternal factors associated with children’s food preferences were determined using a researcher designed, validated tool through face-to-face interview with mothers. Data were analyzed by SPSS software version 16 using General Linear Model to assess the correlations between different variables.

**Results:**

The children with diploma-educated mothers had fewer preferences in consuming nuts, vegetables, and fruits, and children with academic-educated mothers had fewer preferences in consuming nuts, beans, vegetables, fruits, drinks, condiments, and snacks (P<0.05). Children with employed mothers had fewer preferences for beans and drinks (P<0.02). Drinks preferences were lower among children whose mothers had good nutritional knowledge score (P<0.03). Proteins, beans, fruits, condiments, and snacks preferences were higher among children whose mothers had good nutritional attitude score (P<0.05). A positive correlation was found between the food preferences of children and mothers (0.377<B<0.570, P<0.001) in all food groups.

**Conclusion:**

The results of the study showed that mothers̓ educational level and their high nutritional knowledge and attitude could not guarantee the healthy dietary patterns of children. To promote children’s dietary patterns, it is imperative to improve the food preferences of mothers through specialized training.

## Introduction

Healthy nutrition in the early years of life has great importance since it can provide growth, and development needs of children and influence their health in later years of life [1]. Inadequate and unbalanced nutrition in childhood may lead to wasting, stunting, anemia [2], obesity [3], cardiovascular disorders, type 2 diabetes, high blood pressure, and cancer [4]. Childhood is the most sensitive stage to develop food preferences, and the age range between 1-3 years old is a critical period for the formation of these preferences [5]. Food preferences result from the complex interaction of biological trends and environmental influences [6]. Development of food preferences begins from gestation period [7] and continues in childhood, adolescence [8], and adulthood [9]. Therefore, childhood can be considered a critical period for the establishment of healthy food preferences influencing their health throughout life [10]. In general, children’s diets do not match the nutritional recommendations [11]. Over the past few decades, the consumption of high-energy foods, non-nutritious foods, and sweet drinks has been increased, while intake of nutritious foods (e.g., fruits, vegetables, and whole grains) has been decreased [12]. Nowadays, families have fewer meals at home, and they more often eat outdoors or in restaurants [13]. These foods have higher energy and lower nutritional value [14], and their consumption is associated with variety of chronic diseases and adverse health effects [15]. Generally, parents have the main role in the development of dietary habits in children. Parents can create an environment for children that may either lead to enhancement of the healthy eating habits or causing negative effects on their nutrition, which in turn increases unhealthy dietary behaviors and weight gain in children [16]. Besides, nutritional knowledge and attitude of parents may influence the quality of children’s diet. For example, some studies have reported that knowledge and dietary attitude of parents were correlated with the children’s preferences to consume vegetables, fruits [17], fish [18], and fats [16]. Educational level, occupational status, and income level of parents, especially mother, are other factors influencing the nutrition of children as well [19]. Mothers, whether employed or not, spend more time with their children rather than fathers [20] and usually have significant responsibility for planning, purchasing, and preparing the food [21]. A better understanding of the correlation between parental factors and children’s nutrition is vital in particular for the development of supportive interventions. To our knowledge, there are a limited number of studies in the literature regarding potential effects of parental determinants such as socioeconomic factors and food preferences on children’s diet and food preferences, and most of them have been conducted in western societies. Thus, the current study was aimed to investigate the correlation between maternal factors related to their food preferences including their education level, occupational status, as well as their nutritional knowledge and attitude on children’s food preferences in Iran.

## Materials and Methods

In this cross-sectional study, a total of 576 healthy children aged 3-6 years through simple cluster sampling method recruited from nursery schools of all regions, in Tabrizcity, Iran, April 2018 to March 2018. The exclusion criteria were having any illnesses including chronic, psychological, allergic disorders, and any special diet. The study procedure was approved by the Ethics Committee of Tabriz University of Medical Sciences (approval code: IR.TBZMED.REC.1397.236), and the informed consent was obtained from mothers before enrollment in the study.

### Questionnaires

In this study, a four-part questionnaire was used, which included general information of children and mothers, mother’s food preferences, children’s food preferences, and nutritional knowledge and attitude of mothers. Either of food preferences questionnaires for children and mothers had 160 items for ten food groups including proteins, breads and cereals, nuts, beans, dairy, oils, drinks, vegetables, fruits, condiments, and snacks. A 5-point Likert scale was used in the food preferences questionnaires. The items were scored from 1 to 5 and expressed respectively as: “Does not like at all, does not like, neither likes nor dislikes, likes, and likes very much.” Mother’s nutritional knowledge questionnaire included 14 multiple-choice questions, and the nutritional attitude of mother included 30 questions with a 4-point Likertscale ranging between strongly disagree to strongly agree.

### The Validity of the Questionnaires

To assess the validity of the questionnaires, all items were assessed by a panel of nutrition experts (n=15). To evaluate the Content Validity Index (CVI), three criteria of simplicity, relevance, and clarity were used. The items were accepted if each item had a CVI higher than 0.79. In this study, CVI was obtained as 0. 97. To determine the Content Validity Ratio (CVR), the experts rated using three options including necessary, useful but unnecessary, and unnecessary. The cut-off point for CVR was equal to 0.49, and an average of 0.95 was obtained for CVR of the questionnaires. To assess the formal validity, in qualitative stage, the panel of experts rated for apparent validity, logic, comprehensibility, appropriateness, interesting and logical appearance of items, abbreviations, comprehensiveness, writing, and eloquence of instrument items. In the quantitative stage, to reduce and eliminate the inappropriate items, and determine the importance of each item, the item impact formula was used. If the score of item responded by the experts was more than 1.5, the item was confirmed; otherwise, the item was deleted [22].

### Reliability of the Questionnaires

To determine the reliability of the study, 30 children and their mothers were randomly selected, and questionnaires were completed through a face-to-face interview with mothers. Cronbach’s alpha coefficient was calculated as 0.95. These participants were excluded from the original study. According to the results, three identical questions did not earn points from the CVI, CVR, and formal validity. Also, according to the views-points of nutritionists, five questions were combined with other questions. The final questionnaires included 155 items for both mother ʼs and children’s food preferences, 14 items for maternal nutritional knowledge questionnaire, and 28 items for maternal nutritional attitude questionnaire.

### Data Collection

All questionnaires were completed by the same researcher through face-to-face interviews with mothers. To avoid bias in the reports, the researcher who interview with mothers individually, and prior to the interview, given a full explanation to them about the importance of correctly answering the questionnaire.

### Statistical Analysis

Data analysis was performed using SPSS software 16 (IBM, Chicago, IL, USA). Qualitative variables were reported as frequency and percentage, and quantitative variables were presented as mean and standard deviation. Normality for quantitative variables was investigated using the Kolmogorov–Smirnov test. Univariate and multivariate analyses were applied using the General Linear Model (GLM) to determine the possible correlation between different variables. Qualitative variables were entered into the model as dummy variables. A P-value of <0.05 was considered as statistically significant.

## Results

### Demographics Data

Table-1 shows the demographic characteristics of the mother and their children. In this study, a total of 576 children along with their mothers participated, among which 52.6 % (303) of children were girls. The mean age of children and mothers were 5.19 ± 0.920 and 33.82 ± 4.926 years, respectively. About half of the mothers (51%) had an academic degree, and 76.2% of them were housewives.

### Food Preferences of Children and Mothers and Their Correlation

The correlation between mother’s and children’s food preferences are shown in Table-2. The highest and lowest mean score of children’s food preferences belonged to nuts and oils (4.20 ± 0.632, 3.54 ± 0.805, respectively). Regarding the maternal food preferences, the highest mean score belonged to the nuts group (4.42 ± 0.515), and the lowest mean score belonged to the oils group (3.65 ± 0.600). After adjustments for mother’s age, educational level, and occupation status, data analysis using GLM showed that there are statistically significant positive correlations between food preferences of children and their mothers in all food groups (0.377<B<0.570, P<0.001).

### Correlation between Food Preferences of Children and Education Level and Occupation of Mothers

Table-3 shows the correlation between children’s food preferences, mother’s educational level, and occupation status. There were significant negative correlations between diploma and academic educational levels with children’s food preferences in nuts (B= - 0.247; P=0.002 and B= - 0.273, P<0.001, respectively), vegetables (B= - 0.170; P=0.019 and B= - 0.212; P=0.002, respectively) and fruits (B= - 0.137; P=0. 046 and B= - 0.172; P=0.008, respectively). Additionally, mother’s academic educational level and occupation status had significant negative correlation with food preferences in beans (B= - 0.193, P=0.014 and B= - 0.171; P=0.010, respectively) and drinks (B= - 0.251; P<0.001 and B= - 0.207; P=0.001, respectively) and academic educational level of mothers was negatively related with children’s food preferences in condiments and snacks (B= - 0.182, P=0.001). Maternal educational level and occupation status have not a significant correlation with food preferences in proteins, breads and cereals, dairy, and oils. Also, mother’s occupation status have not significant correlation with children’s food preferences in nuts, vegetables, fruits, condiments, and snacks.

### Correlation between Nutritional Knowledge and Attitudes of Mothers with Children’s Food Preferences

Table-4 shows the correlation between nutritional knowledge and attitude of mothers with children’s food preferences. Mean scores of nutritional knowledge and attitude of mothers were 11.59 ± 1.885 and 86.70 ± 7.190, respectively. Children whose mothers had good nutritional knowledge had score= - 0.191 in drinks group, which was fewer than their peers whose mothers had less knowledge (B= - 0.191, P=0.026). Regarding nutritional attitude of mothers and according to GLM results, in children whose mothers had an intermediate level of nutritional attitude, in average, score of food preferences in fruits group was 0.119 higher than children whose mothers had weak attitude (B=0.119, P=0.028). Additionally, in children whose mothers had a good level of nutritional attitude, in average, score of preferences in proteins 0.164 (B=0.164, P=0.001), beans 0.221 (B=0.221, P=0.001), condiments and snacks 0.114 (B=0.114, P=0.015), and fruits 0.200 (B=0.200, P<0.001) were higher compared to children whose mothers had a weak nutritional attitude. Nutritional knowledge and attitude of mothers were not related to food preferences in breads and cereals, nuts, dairy, oils, and vegetables. Nutritional knowledge of mothers was not significantly associated with children’s food preferences in food groups of proteins, beans, fruits, condiments, and snacks; also, the nutritional attitude of mothers was not related to children’s food preferences in drinks.

## Discussion

This study was carried out to identify maternal factors associated with food preferences of children aged 3-6 years. The results of the study showed that the highest score of food preferences in mothers and children was related to nuts and the lowest score was related to oils, and there was a positive correlation between mothers’ food preferences in all food groups and children’s food preferences. Findings of this study were consistent with previous studies, indicating the correlation between mothers’ and children’s food preferences [23, 24]. However, in other studies, this correlation has been reported between some, but not all food groups. For example, Vereecken *et al*. showed that the consumption of fruits, vegetables, and sweet drinks was similar between mothers and their children aged 2.5-7 years [25]. Various studies have shown that consumption of fruits and vegetables by the mothers are the strongest predictor of children’s preference in fruit and vegetables in preschool [26]. Palfreyman *et al*. also revealed a positive correlation between consumption of snacks by mothers and children [27]. Therefore, to improve children’s food preferences, it is advisable for mothers to have healthy and diverse diets regardless of their dietary preferences [28]. In this study, mothers̓ education level (diploma and academic) was negatively related with children’s food preferences in nuts, vegetables, and fruits, and also mother’s academic educational level was negatively correlated with children’s food preferences in beans, drinks, condiments, snacks. Previous studies revealed that mothers’ educational level has the greatest effect on the nutritional status of children [29]. Mothers with lower educational level have fewer healthy eating habits and less fruit and vegetable consumption compared to mothers with high one [30], and mothers̓ low educational level is associated with consumption of drinks, juices with added sugar, and other unhealthy foods [31]. Children whose mothers have a higher level of education prefer to consume water, rice, bread, vegetables, fruits, meat, fish, poultry, egg, and dairy products more than those whose mothers have a moderate or lower level of education [32]. Our findings regarding food preferences of children with academic-educated mothers in drinks, condiments, and snacks were similar to other studies, but they were inconsistent with previous studies regarding food groups of cereals, fruits, and vegetables. Generally, the desire to prepare foods in fast and easy ways can lead to the decreased consumption of nutritious foods and healthy diets by children [33], in particular, employed mothers are more likely not to devote enough time for food preparation [34]. Mothers who are employed are paying less attention to food prices while shopping [35]. In the present study, children with employed mothers had fewer preferences in beans and drinks compared to children of housewife mothers. Our result regarding preferences in beans was consistent with previous studies, but, it was contrasted to previous studies with respect to preferences in drinks. The results of this study revealed that mothers’ good level of knowledge had a negative correlation with food preferences in drinks. Mothers’ moderate level of nutritional attitude had positive correlation with food preferences in fruits. Children whose mothers had good nutritional attitude had more preferences in proteins, beans, condiments and snacks, and fruits. Previous studies have shown that family members, especially mothers, are the most important source of nutrition education [36]. Mothers with higher nutritional knowledge have been shown to give their children more cheese and eggs [32]. High nutritional knowledge of mothers has a negative relation with the consumption of snacks by their children [37]. Nutritional knowledge and attitude of mothers have a positive correlation with the consumption of fruits and vegetables [17], and the consumption of fish [18] and have a negative correlation with the intake of fats [16]. In this study, children whose mothers had good nutritional knowledge preferred to drink less, and children whose mothers had good nutritional attitude preferred to consume more proteins, beans, and fruits. Our results were in line with the previous studies. Children’s food preferences in condiments and snacks were higher in mothers with good nutritional attitude preferences score, which contrasted with the previous studies.

### Limitations and Strengths

Due to the low age of the children participated in the study, the children’s food preferences questionnaire was completed by the researcher through face-to-face interviews with their mothers. This could potentially influence the accuracy of the reported data, to some extent. However, the present study was the first study that investigated the correlation between maternal factors related to children’s food preferences using validated tools in Iran.

## Conclusion

This study presented new and surprising information about the correlation between maternal factors and children’s food preferences. Due to the similarity between mother’s and children’s food preferences, mothers need to improve their food preferences and make healthy and nutritious food available to their offspring. The results of the study showed that mothers̓ educational level and their high nutritional knowledge and attitude alone does not influence on improving the nutritional status of children. Thus, specialized and ongoing training in relation to nutrition for mothers as well as understanding the effect of mother’s food preferences on children’s food preferences can lead to healthy eating habits.

## Acknowledgment

Authors thankful of the Welfare Organization, directors of nursery schools, and mothers and their children who participate in the study.

## Conflict of Interest

There are no conflicts of interest.

**Table 1:** Individual information of children and mothers

**Age of children (years)** ^a^	5.19 ± 0.920
Girl^b^	303(52.6%)
Boy^b^	273(47.4%)
**Age of mothers (years)** ^a^	33.82 ± 4.926
**Mother’s educational level**	
Low-educated^b^	94(16.4%)
Diploma education^b^	188(32.6%)
College education^b^	294(51%)
**Mother’s employment status**	
Employed^b^	137(23.8%)
Housewife^b^	439(76.2%)

**a:** Mean and standard deviation **b:** Frequency and percentage

**Table 2:** Correlation of mother’s food preferences with Children’s food preferences

**Variables**	**Mother’s food preferences**		**Children’s food preferences**		
	**Mean (SD)**	**Mean (SD)**	**B**	**SE**	*P
**Proteins**	4.02 (0.425)	3.84 (0.463)	0.470	0.041	<0.001
**Breads and cereals**	3.95 (0.418)	3.83 (0.429)	0.465	0.038	<0.001
**Nuts**	4.42 (0.515)	4.20 (0.632)	0.464	0.048	<0.001
**Beans**	4.01 (0.503)	3.64 (0.652)	0.504	0.049	<0.001
**Dairy**	3.87 (0.619)	4 (0.695)	0.377	0.045	<0.001
**Oils**	3.65 (0.600)	3.54 (0.805)	0.436	0.054	<0.001
**Drinks**	3.92 (0.536)	4 (0.601)	0.399	0.043	<0.001
**Vegetables**	4.13 (0.446)	3.61 (0.563)	0.485	0.049	<0.001
**Fruits**	4.26 (0.445)	4.09 (0.538)	0.570	0.045	<0.001
**Condiments and snacks**	3.75 (0.499)	3.89 (0.442)	0.387	0.033	<0.001

Dependent variable: Children’s food preferences average scores Findings were obtained using GLM by modifying mother’s age, education level, and occupation.

**Table 3:** Correlation between the level of education and occupation of mothers and children’s food preferences. (n=576)

**Variables**	**Mother’s education level**			**Mother’s employment status**	
	**low-educated (reference)** **94 (16.4%)**	**Diploma education** **188 (32.6%)**	**College education** **294 (51%)**	**Housewife** **(reference )** **439 (76.2%)**	**Employed** **137 (23.8%)**
**Proteins**	B	-	-0.062	-0.085	-	-0.075
	SE	-	0.060	0.056	-	0.047
	P*	-	0.296	0.128	-	0.113
**Breads and cereals**	B	-	-0.076	-0.071	-	-0.078
	SE	-	0.055	0.052	-	0.044
	P*	-	0.173	0.175	-	0.076
**Nuts**	B	-	-0.247	-0.273	-	-0.006
	SE	-	0.081	0.076	-	0.065
	P*	-	0.002	<0.001	-	0.923
**Beans**	B	-	-0.105	-0.193	-	-0.171
	SE	-	0.083	0.078	-	0.066
	P*	-	0.209	0.014	-	0.010
**Dairy**	B	-	-0.093	-0.149	-	-0.043
	SE	-	0.089	0.084	-	0.071
	P*	-	0.299	0.075	-	0.545
**Oils**	B	-	-0.092	0.089	-	0.057
	SE	-	0.103	0.097	-	0.083
	P*	-	0.374	0.359	-	0.486
**Drinks**	B	-	-0.137	-0.251	-	-0.207
	SE	-	0.076	0.072	-	0.061
	P*	-	0.073	<0.001	-	0.001
**Vegetables**	B	-	-0.170	-0.212	-	0.006
	SE	-	0.072	0.068	-	0.058
	P*	-	0.019	0.002	-	0.913
**Fruits**	B	-	-0.137	-0.172	-	-0.053
	SE	-	0.069	0.064	-	0.055
	P*	-	0.046	0.008	-	0.333
**Condiments and snacks**	B	-	-0.063	-0.182	-	-0.081
	SE	-	0.056	0.053	-	0.045
	P*	-	0.264	0.001	-	0.073

* Findings were obtained using GLM by modifying mother’s age.

**Table 4:** Correlation between the nutritional knowledge and attitude of mothers and children’s food preferences (n=576)

**Variables**	**Mother’s Nutritional Knowledge**			**Mother’s Nutritional Attitude**		
	**Weak** **222 (38.5%)**	**Medium** **285 (49.5%)**	**Good** **69 (12%)**	**Weak** **202 (35%)**	**Medium** **198 (34.4%)**	**Good** **176 (30.6%)**
**Proteins**	B	-	-0.037	0.074	-	0.027	0.164
	SE	-	0.044	0.067	-	0.047	0.049
	P*	-	0.405	0.269	-	0.561	0.001
**Breads and cereals**	B	-	0.017	0.046	-	0.050	0.084
	SE	-	0.041	0.062	-	0.044	0.046
	P*	-	0.679	0.457	-	0.259	0.070
**Nuts**	B	-	-0.061	-0.098	-	-0.004	0.106
	SE	-	0.060	0.091	-	0.065	0.067
	P*	-	0.312	0.283	-	0.954	0.115
**Beans**	B	-	-0.024	0.054	-	0.025	0.221
	SE	-	0.062	0.094	-	0.066	0.069
	P*	-	0.692	0.567	-	0.705	0.001
**Dairy**	B	-	0.024	0.091	-	0.052	0.077
	SE	-	0.066	0.100	-	0.071	0.074
	P*	-	0.718	0.365	-	0.463	0.304
**Oils**	B	-	-0.005	0.038	-	0.036	0.085
	SE	-	0.076	0.116	-	0.083	0.086
	P*	-	0.951	0.746	-	0.666	0.324
**Drinks**	B	-	-0.011	-0.191	-	0.014	-0.011
	SE	-	0.056	0.085	-	0.061	0.064
	P*	-	0.840	0.026	-	0.815	0.861
**Vegetables**	B	-	-0.028	-0.039	-	0.036	0.063
	SE	-	0.053	0.081	-	0.058	0.060
	P*	-	0.595	0.633	-	0.532	0.293
**Fruits**	B	-	-0.054	-0.056	-	0.119	0.200
	SE	-	0.051	0.077	-	0.054	0.057
	P*	-	0.286	0.463	-	0.028	<0.001
**Condiments and snacks**	B	-	0.009	0.057	-	0.036	0.114
	SE	-	0.042	0.063	-	0.045	0.047
	P*	-	0.822	0.371	-	0.427	0.015

*Findings were obtained using GLM by modifying mother’s age, education level
